# Hormonal induction of gamete release, and in-vitro fertilisation, in the critically endangered Southern Corroboree Frog, *Pseudophryne corroboree*

**DOI:** 10.1186/1477-7827-8-144

**Published:** 2010-11-29

**Authors:** Phillip G Byrne, Aimee J Silla

**Affiliations:** 1 School of Biological Sciences, Monash University, Melbourne Vic, Australia; 2 School of Animal Biology, University of Western Australia, Perth, Australia

## Abstract

**Background:**

Conservation Breeding Programs (CBP's) are playing an important role in the protection of critically endangered anuran amphibians, but for many species recruitment is not successful enough to maintain captive populations, or provide individuals for release. In response, there has been an increasing focus on the use of Assisted Reproductive Technologies (ART), including the administration of reproductive hormones to induce gamete release followed by *in vitro *fertilisation. The objective of this study was to test the efficacy of two exogenous hormones to induce gamete release, for the purpose of conducting *in vitro *fertilisation (IVF), in one of Australia's most critically endangered frog species, *Pseudophryne corroboree*.

**Methods:**

Male frogs were administered a single dose of either human chorionic gonadotropin (hCG) or luteinizing hormone-releasing hormone (LHRHa), while female frogs received both a priming and ovulatory dose of LHRHa. Spermiation responses were evaluated at 3, 7, 12, 24, 36, 48, 60 and 72 h post hormone administration (PA), and sperm number and viability were quantified using fluorescent microscopy. Ovulation responses were evaluated by stripping females every 12 h PA for 5 days. Once gametes were obtained, IVF was attempted by combining spermic urine with oocytes in a dilute solution of simplified amphibian ringer (SAR).

**Results:**

Administration of both hCG and LHRHa induced approximately 80% of males to release sperm over 72 h. Peak sperm release occurred at 12 h PA for hCG treated males and 36 h PA for LHRHa treated males. On average, LHRHa treated males released a significantly higher total number of live sperm, and a higher concentration of sperm, over a longer period. In female frogs, administration of LHRHa induced approximately 30% of individuals to release eggs. On average, eggs were released between 24 and 48 h PA, with a peak in egg release at 36 h PA. IVF resulted in a moderate percentage (54.72%) of eggs being fertilised, however all resultant embryos failed prior to gastrulation.

**Conclusions:**

Hormone treatment successfully induced spermiation and ovulation in *P. corroboree*, but refinement of gamete induction and IVF techniques will be required before ART protocols can be used to routinely propagate this species.

## Background

Environmental change driven by anthropogenic activities is causing unprecedented rates of species extinction, presenting a major threat to global biodiversity [1]. Among vertebrates, all classes have suffered high extinction rates, but amphibians have been most severely impacted. Based on recent estimates, more than one-third of the worlds amphibians are threatened with extinction [2,3], and almost one-half of the remaining species are in a state of decline [2]. In response to this crisis, the international Amphibian Conservation Action Plan (ACAP), devised in 2005, urged the establishment of captive assurance colonies for threatened species [4,5]. In accordance with this recommendation, a large number of institutions worldwide have initiated captive breeding programmes for declining and endangered amphibians. However, in almost all cases, breeding attempts have failed due to the inherent difficulties associated with simulating the complex combination of social and environmental factors that trigger amphibians to breed [6,5]. In reaction to this captive breeding crisis, there has been a growing interest in determining whether threatened amphibians can be propagated, and genetically managed, using assisted reproductive technologies (ART)[5,7,8].

One component of ART is the artificial manipulation of reproductive events using exogenous hormones. Specifically, males and females are administered hormones to stimulate the production and release of gametes (spermatozoa and oocytes), which are then used to generate embryos via *in vitro *fertilisation (IVF), also referred to as artificial fertilisation (AF) [5]. Among anurans (frogs and toads), it has been known for several decades that exogenous gonadotropins, and gonadotropin-releasing hormones, can be used to induce both sperm release (spermiation) and oocyte release (ovulation) [9-12]. Pituitary extracts are an effective source of amphibian gonadotropins and such preparations have been successfully used to induce gamete release in various anuran species, including *Bufo arenarum*, *Rana pipiens*, *Hyla regilla *and *Eleutherodactylus coqui *[9,10,13-16]. However, the use of pituitary extracts is now strongly discouraged due to a high risk of pathogen transmission [5,7], and because production of pituitary preparations requires the euthanasia of large numbers of reproductively mature anurans [17]. An alternative approach is the use of synthetic hormones, in particular, commercially available analogues of luteinizing-hormone releasing hormone (LHRH) and human chorionic gonadotropin (hCG) [8,17-19].

Luteinizing-hormone releasing hormone is a hypothalamic hormone that acts by stimulating the anterior pituitary to synthesize and release natural luteinizing hormone (LH), which in turn stimulates gonadal activity. In contrast, hCG acts by mimicking LH, due to identical alpha and shared beta subunits [20], bypassing the hypothalamic-pituitary-gonadal axis to exert a direct influence on the gonads [21]. Evidence that synthetic analogues of LHRH and hCG can successfully induce gamete release has been obtained for a broad diversity of anuran species [7,17,18,22-26], but the efficacy of these hormones has been found to vary considerably. For example, LHRHa is significantly more effective than hCG at stimulating ovulation in *Eleutherodactylus coqui *[17], but hCG is highly effective at stimulating ovulation in *Xenopus laevis *[26].

Although there is still much to learn about the relative efficacy of hCG and LHRHa across species, baseline knowledge concerning their potency has permitted IVF to be attempted in a small, but growing number of anuran families, including the bufonidae [8], pipidae [26] and myobatrachidae [27]. Surprisingly, however, almost all anuran IVF studies conducted to date have refrained from artificially fertilizing oocytes using hormonally induced sperm, instead opting to use sperm obtained from testes macerates [13,27-29]. The benefit of conducting IVF using testes macerates is that sperm can be obtained in high concentrations, and can also be acquired at the exact moment when females begin ovulating, eliminating the need for sperm storage [19]. Given these practical advantages, euthanizing males for the purpose of IVF might be useful in common species, but this approach cannot be justified in endangered species where individual animals are of high genetic value [5,7].

To date, few studies have attempted IVF in anurans using hormonally induced gametes collected from live animals, and outcomes have been highly variable. For example, Waggener and Carrol [22] achieved 100% fertilisation success in the leptodactylid frogs *Lepidobatrachus laevis *and *L. illanensis *[22], but Browne et al. [8] reported a mean fertilisation success of 12.7% in the endangered toad *Bufo baxteri*. This extreme variance in IVF success, which is probably related to species-specific differences in reproductive mode and physiology, suggests that protocols for combining hormonally induced gametes are not readily transferable between species [19]. Given this inherent level of unpredictability, there is a need to develop techniques for collecting and combining gametes obtained from live individuals. In particular, there is an urgent need to develop these protocols for endangered species [7,8].

The southern corroboree frog *Pseudophryne corroboree*, is one of Australia' s most critically endangered frog species [30]. Of the species known to be extant in Australia, *P. corroboree *was ranked by the Australasian Regional Association of Zoological Parks and Aquaria as the highest priority anuran requiring ex-situ conservation [31]. The distribution of *P. corroboree *is highly restricted, with the species confined to a linear distance of 51 km within subalpine regions of Koscuiszko National Park [32]. According to recent field surveys, there are currently less than 50 individuals remaining at natural breeding sites, and the species is predicted to go extinct in the wild within the next ten years [33]. Population declines in *P. corroboree *were first observed in the 1980's, and since 1996 the species has been the focus of an intensive management and recovery programme [33,34]. Early management of the species concentrated on habitat protection, but more recently efforts have turned towards the establishment of captive assurance populations [33,35]. At present, multiple populations of *P. corroboree *are being maintained in zoos and biological institutions throughout Australia, but successful breeding and recruitment in captivity has been limited. Despite this critical situation, there has been no attempt to bolster captive breeding activity using assisted reproductive technologies.

The aim of this study was twofold. First to test the efficacy of using LHRH and hCG to induce spermiation, and LHRH to induce ovulation, in captively reared *P. corroboree*, and second, to test whether hormonally induced sperm and oocytes can be used to generate embryo's via *in-vitro *fertilisation (IVF).

## Methods

All research was conducted in compliance with the Monash University Animal Ethics Committee (AEC), permit number BSCI/2009/27.

### Study population

Frogs were obtained from a captive colony maintained at the Amphibian Research Centre (ARC) in Melbourne Victoria. The captive colony was established by collecting natural egg clutches from multiple populations throughout the species range during the 2004 and 2005 breeding seasons. The eggs were reared to maturity and housed in indoor terrariums (150 × 43 × 38 cm) exposed to seasonal fluctuations in temperature and photoperiod that mimicked natural conditions. Frogs used in this study were approximately 4-5 years old because sexual maturity in *P. corroboree *is not reached until 3-4 years post-metamorphosis [36]. In total, the study involved 24 males and 25 females, but one male was not treated. In *P. corroboree *phenotypic traits do not provide reliable indicators of sex. Therefore, prior to commencement of the study all frogs were genetically sexed using Amplified Fragment Length Polymorphism (AFLP) analysis, with the presence of a 66 bp DNA marker (representing a Y chromosome sequence) diagnostic for males [37,38].

Frogs were collected from the ARC and transported to Monash University (Clayton campus) where they were held for the duration of the study (January 29-April 9, 2009). On the day of collection, frogs were weighed and snout-vent length measured before being randomly assigned to same sex groups (n = 6 frogs per group, with the exception of one female group that contained 7 frogs). Groups of individuals were housed in plastic enclosures (360 mm × 200 mm × 170 mm), each containing a layer of fine gravel (~15 cm thick) covered with a layer of sphagnum moss (~5 cm thick). Once a week, containers were flushed with approximately 2 L of deioinized water and frogs were fed ten-day old crickets (~200 per container). All containers were kept in a constant temperature room maintained on a 17°C/12°C day/night temperature cycle and a 14.5 h/9.5 h light/dark cycle.

### Hormonal induction of spermiation

Males were administered a single dose of either 20 IU per gram bodyweight hCG (chorolon^®^) (n = 6 males) or 5 μg per gram bodyweight LHRHa *(Leuprorelin oxo-Pro-His-Trp-Ser-Tyr-[d-leu]-Leu-Arg-Pro-NHEt*: Lucrin^®^) (n = 11 males). These doses were selected because they approximate doses previously found to induce gamete release in anurans [5,22,24,39]. Hormones were diluted in 100 μL of Simplified Amphibian Ringer (113 mM NaCl, 2 mM KCl, 1.35 mM CaCl_2_, 1.2 mM NaHCO_3_) and administered via subcutaneous injection into the dorsal lymph sac. As a control for the injection and handling procedures, a third group of frogs (n = 6) were administered 100 μL of Simplified Amphibian Ringer (SAR). Following hormone administration, frogs were returned to plastic holding tanks (50 mm × 90 mm) containing moist sponge hydrated with 5 mL of distilled water. Under these conditions, frogs were sufficiently hydrated to permit urine collection at each of the sampling times.

Spermic urine was collected at 3, 7, 12, 24, 36, 48, 60 & 72 h post hormone administration (PA). The collection method involved gently inserting the end of a glass microcapillary tube (fire polished and cooled) into the cloaca to stimulate urination. Immediately post collection, the volume of urine collected was measured in microlitres (μL), and the sample was then homogenized with 5 μL of a 1:50 dilution of SYBR-14 (Invitrogen L-7011), and then incubated in the dark for 7 min. Following this, a 2 μL aliquot of propidium iodide (PI) was then added and the solution was incubated in the dark for a further 7 min. SYBR-14 and PI are membrane permanent DNA stains that are commonly used to quantify sperm number and viability in anurans [39,40] and other vertebrates [38]. SYBR-14 specifically stains the DNA of live (viable) sperm, while PI specifically stains the DNA of dead (non-viable) sperm. Under UV light, live sperm (stained with SYBR-14) fluoresce bright green, while dead sperm (stained with PI) fluoresce bright red [41]. Immediately after staining, wet mount slides were prepared and the viability of sperm was evaluated within 30 min using fluorescent microscopy at a wavelength of 490 nm. For each sample, we calculated the total sperm count, sperm concentration (number of sperm/urine volume (μL) × 1000), and sperm viability (the proportion of live/total sperm).

### Hormonal induction of ovulation

Females were randomly allocated to one of two treatment groups; a hormone treatment (n = 17 females) administered LHRHa (Lucrin^®^), or a control treatment (n = 8 females) administered Simplified Amphibian Ringer (SAR). For the hormone treatment, a stock solution of Lucrin^® ^was diluted in Simplified Amphibian Ringer to produce a final concentration of 100 μg mL^-1^. Females received an anovulatory dose of 1 μg LHRHa per gram bodyweight diluted in 100 μL of SAR administered via subcutaneous injection into the dorsal lymph sac. This dosage was administered to prime the ovary without inducing ovulation [8,42]. Twenty-six hours after administration of the 'priming dose', each female received an ovulatory dose of 5 μg LHRHa per gram bodyweight. For the control treatment, females were administered 100 μL of SAR in place of the priming and ovulatory doses. Following treatment, individual females were placed into plastic enclosures (200 mm × 120 mm × 90 mm) lined with moist sponge and sphagnum moss. Individual animals were removed from their holding tanks 12 h PA of the ovulatory dose and stripping (expulsion of eggs from the oviducts) was attempted. Stripping was facilitated by holding a frog with its legs extended and gently applying pressure to the abdomen in a craniocaudal direction [21,43]. Stripping was attempted every 12 h ± 0.5 h for a period of five days.

### *In-vitro *fertilisation (IVF)

At each sampling time, any eggs expelled from a female were placed in an individual dry Petri dish and IVF was conducted using available spermic urine samples. An aliquot of approximately 170 μL of pooled spermic urine was activated in approximately 100 μL 1:4 SAR. Sperm concentrations used for IVF ranged between 1.14 × 10^2 ^and 2.87 × 10^2^. The sperm solution was pipetted directly onto the oocytes and the dish was agitated for one minute. Each dish was enclosed within a petri dish and left to develop in a constant temperature room set to 10°C. Developing embryos were supplied with 100 μL of deionised water at 12 h, and a further 1000 μL at 24 h post fertilisation. Fertilisation success was calculated as the proportion of eggs at Gosner stage 4 to 6 [44] approximately 12 h post application of spermic urine to the oocytes. Embryonic development was checked every 6-12 h for a period of 7 days, and developmental stage quantified, using a stereo dissecting microscope.

### Statistical analyses

The number of males that released sperm was compared between experimental treatments (LHRHa versus hCG), and between each experimental treatment and the control (hCG versus control, LHRHa versus control), using Fisher's exact tests. All comparisons were one-tailed due to the expectation of a positive treatment response. Comparison of mean total sperm count and mean total sperm concentration over the 72 h sampling period was made between treatments using Welch ANOVA's, due to unequal variance. Calculations of total sperm concentration only included sampling times in which sperm were released. Comparisons of sperm count and sperm concentration over time were made between treatments using repeated measures MANOVA's, with the main factors set as hormone treatment, and the within subject factors set as time. The MANOVA's were based on 6 equally spaced time intervals: 12 h, 24 h, 36 h, 48 h, 60 h and 72 h PA. Because the data violated the assumption of sphericity (Mauchly's test: P < 0.05), univariate analyses were corrected, and degrees of freedom adjusted, using the Greenhouse-Geisser method. The mean proportion of live sperm released over 72 h was compared between treatments using a student t-test. For all spermiation analyses, sperm counts were square root transformed, and sperm proportions were arcsine transformed. The number of females ovulating in response to hormone treatment (LHRHa injection) was compared to the control treatment (saline injection) using a one-tail Fisher's exact test. All statistical comparisons were performed using JMP software, with significance levels set at *P < 0.05*.

## Results

### Hormonal induction of spermiation

**Figure 1:**
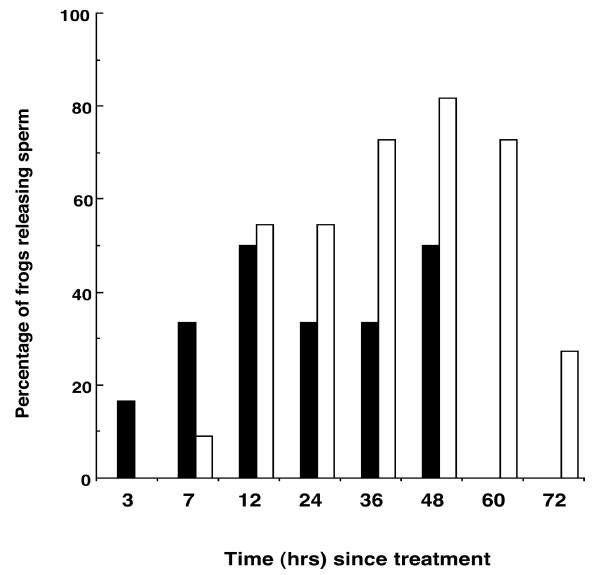
**Percentage of hormone treated males releasing sperm over a 72 h sampling period**. Frogs were administered either hCG (n = 6 males)(black bars) or LHRHa (n = 11 males)(white bars).

No frogs (0/6) released sperm following injection of a saline control, but a high proportion of males released sperm following injection of hCG (83.3%, 5/6) and LHRHa (81.8%, 9/11). The number of males releasing sperm was significantly higher in response to hormone treatment compared to the control treatment (control versus hCG, Fisher's exact test: p = 0.007; control versus LHRHa, Fisher's exact test p = 0.002), but the number of responding males did not significantly differ between hormone treatments (hCG versus LHRHa, Fisher's exact test: p = 0.72). Males treated with hCG started releasing sperm within 3 hrs post administration (PA), and ceased sperm release after 48 h PA (Figure 1). In contrast, males treated with LHRHa, did not commence sperm release until 7 h PA, and at 72 h PA over 25% (3/11) of males were still releasing sperm (Figure 1). The highest proportion of males releasing sperm occurred between 12 and 48 h in response to hCG, and between 36 and 60 h in response to LHRHa (Figure 1).

**Figure 2:**
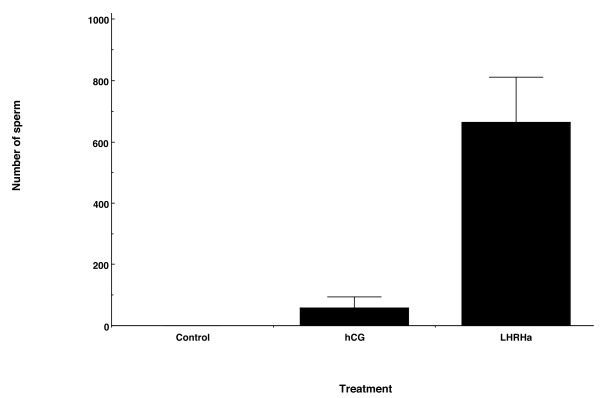
**Total number of sperm released by hormone treated males over a 72 h sampling period**. Values represent mean ± SEM total number of spermatozoa released following subcutaneous injection of i) saline solution (control, n = 6 males) ii) hCG (n = 6 males) or iii) LHRHa (n = 11 males).

The mean total number of sperm released over 72 h was more than 11 times higher in LHRHa treated males than in hCG treated males (Figure 2), and this difference was significant (Welch's ANOVA; F_1, 11.06 _= 11.28, P = 0.006). There was no overall time effect on the number of sperm released (MANOVA: F _3.01, 36.15 _= 1.42, p = 0.46) and no significant interaction between time and treatment (MANOVA: F _3.01, 36.15 _= 1.05, p = 0.38), indicating that the number of sperm released did not differ between treatment groups over time. However, there was a significant treatment effect (MANOVA: F _1,12 _= 8.14, p = 0.01), indicating that at each sampling period, LHRHa treated males generally released more sperm than hCG treated males (Table 1). Peak sperm release occurred at 12 h PA for hCG treated males, but not until 36 h PA for LHRHa treated males (Table 1).

**Table 1:** Number of sperm, sperm concentration and sperm viability of sperm released by males between 3 and 72 h post administration of hCG (n = 6) and LHRHa (n = 11)

Treatment	Time (PA)	Sperm number	**Sperm/mL (× 10**^**3**^**)**	Sperm viability
hCG	3	1.20 ± 1.20	0.086 ± 0.086	1.00 ± 0.00
hCG	7	0.40 ± 0.20	0.009 ± 0.005	1.00 ± 0.00
hCG	12	39.0 ± 38.25	1.72 ± 1.703	0.34 ± 0.32
hCG	24	5.00 ± 4.75	0.16 ± 0.147	0.85 ± 0.14
hCG	36	2.20 ± 1.95	0.27 ± 0.263	0.60 ± 0.40
hCG	48	9.20 ± 5.34	0.03 ± 0.022	0.00 ± 0.00
hCG	60	0.00 ± 0.00	-	-
hCG	72	0.00 ± 0.00	-	-
LHRHa	3	0.00 ± 0.00	-	-
LHRHa	7	17.22 ± 17.22	1.111 ± 1.111	0.68 ± 0.00
LHRHa	12	101.55 ± 67.28	3.113 ± 1.837	0.89 ± 0.03
LHRHa	24	82.33 ± 47.93	3.507 ± 1.937	0.80 ± 0.08
LHRHa	36	233.88 ± 99.73	10.106 ± 4.018	0.85 ± 0.02
LHRHa	48	149.00 ± 67.42	0.776 ± 0.368	0.72 ± 0.04
LHRHa	60	67.00 ± 39.16	0.313 ± 0.162	0.30 ± 0.05
LHRHa	72	12.44 ± 6.65	0.733 ± 0.389	0.54 ± 0.24

Values represent means ± SEM

**Figure 3:**
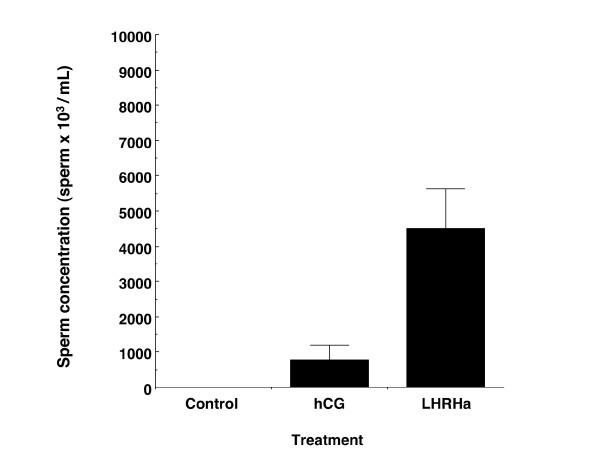
**Total concentration of sperm released by hormone treated males over a 72 h sampling period**. Values represent mean ± SEM total concentration of spermatozoa released following subcutaneous injection of i) saline solution (control, n = 6 males) ii) hCG (n = 6 males) or iii) LHRHa (n = 11 males).

Mean total sperm concentration was significantly higher in LHRHa treated males than in hCG treated males (Welch's ANOVA; F_1, 11.70 _= 9.33, p = 0.01, Figure 3). Sperm concentration did not significantly differ over time (MANOVA: F _2.19, 26.32 _= 2.68, p = 0.082), and there was no significant interaction between time and treatment (MANOVA: F _2.19, 26.32 _= 1.79, p = 0.18), indicating that over time sperm concentration did not differ between treatment. However, there was a significant overall treatment effect (MANOVA: F _1, 12 _= 18.31, p = 0.001), indicating that at individual sampling times sperm concentration was generally higher for LHRHa treated males than hCG treated males (Table 1). Peak sperm concentration occurred at 12 h for hCG treated males and 36 h for LHRHa treated males, corresponding with peaks in total sperm number (Table 1).

**Figure 4:**
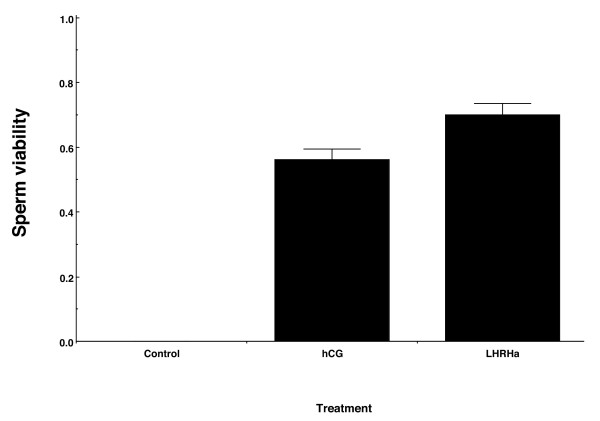
**Total proportion of live sperm released (sperm viability) by hormone treated males over a 72 h sampling period**. Values represent mean ± SEM total proportion of live spermatozoa released by frogs following subcutaneous injection of i) saline solution (control, n = 6 males) ii) hCG (n = 6 males) or iii) LHRHa (n = 11 males).

The mean total proportion of live sperm (sperm viability) released over 72 h, was almost 10% higher in LHRHa treated males than in hCG treated males (Figure 4), and this difference was significant (t-test; t = 2.31, df = 11, p = 0.04). Males treated with hCG released live sperm between 3 and 36 h PA, and over this period sperm viability was generally high (>60%), but at 12 h PA, which was the peak time for sperm release (maximum sperm number and concentration), average viability was lower than 35% (Table 1). For LHRHa treated males, live sperm were released between 7 and 72 h PA, and the sperm viability remained above 60% up until 48 h PA, after which time viability started to drop (Table 1). At the time of peak sperm production and concentration (36 h PA), the proportion of live sperm was higher than 80% (Table 1).

### Hormonal induction of ovulation

**Figure 5:**
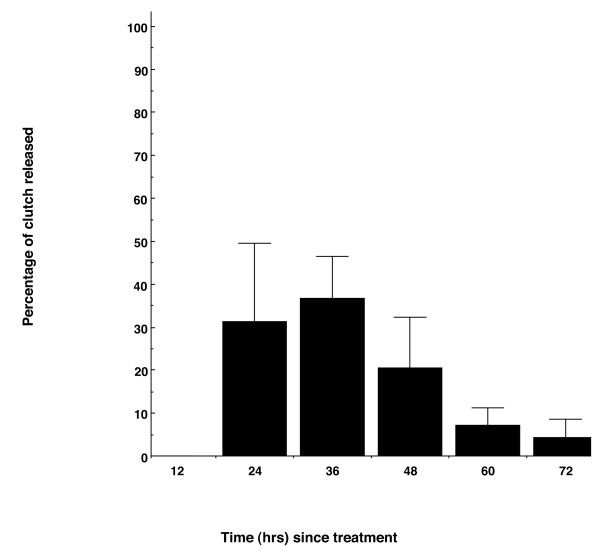
**Percentage of total clutch released by female frogs (n = 5) every 12 h over a 72 h sampling period**.

No frogs (0/8) released eggs following injection of a saline control, and almost 30% (5/17) of females released eggs following injection of LHRHa, but this difference was not significant (Fishers exact test p = 0.116). The mean body size (SVL) of females that released eggs (mean ± SE = 28.3 ± 0.75) was not significantly different from females that did not release eggs (mean ± SE = 27.58 ± 0.48) (t = -0.793, df = 15, p = 0.44). There was also no significant relationship between female body size (SVL) and total clutch size (r^2 ^= 0.473, n = 5, p = 0.199). Of the females that responded positively to hormone treatment, all of them (5/5) released their clutches in discrete batches over 2-4 sampling times (Table 2). Females released between one and twelve eggs per batch, and average total clutch size was 15.2 ± 2.67 (Table 2). No female released eggs until 24 h PA, and only one female was still releasing eggs at 72 h PA (Figure 5). On average, females released the greatest proportion of their clutches between 24 and 48 h PA, with a peak in egg release at 36 h PA (Figure 5).

**Table 2:** Egg release patterns and IVF success for five Pseudophryne corroboree females

Id	Time (PA)	Batch	Total eggs	% Fertilised	% Survival	Developmental stage at failure
3060	24	1	12	75	0	4-6
	36	2	8	100	0	4-6
	48	3	1	-	-	-
	60	4	1	-	-	-
7	24	1	2	-	-	-
	36	2	5	40	0	9-10
	48	3	11	100	0	3-4
	60	4	2	100	0	3-4
4080	36	1	4	25	0	4-6
	48	2	3	0	0	3-4
809	24	1	12	41.7	0	9-10
	36	2	1	0	0	-
2090	36	1	8	100	0	3-4
	60	2	3	33.33	0	4-6
	72	3	3	100	0	3-4

NB: For females 7 and 3060, IVF was not performed at 24 h, 48 h and 60 h respectively (missing values), because eggs were not viable when released

### *In-vitro *fertilisation

On average, fertilisation success across females (n = 5) was moderate (mean ± SE = 54.72 ± 12.80%), but there was considerable variation between females (Table 2). Of the eggs that were fertilised, all commenced embryonic development, but no embryos survived beyond gastrulation [44]. The exact stage of failure varied between a female's egg batches, as well as between females (Table 2).

## Discussion

Our study showed that hormone treatment induced gamete release in both male and female corroboree frogs, permitting us to test whether IVF can be used to augment captive breeding in this critically endangered species. Spermiation was induced following administration of either hCG or LHRHa, but these hormones were not equally effective. Both hormones led to approximately 80% of males releasing sperm, but there were significant differences in the speed and duration of response, and the number, concentration and proportion of live sperm released. Administration of hCG, led to a more rapid response, but LHRHa induced the release of a significantly higher number and concentration of viable sperm, and over a longer time period. This difference indicates that LHRHa was more effective at inducing spermiation in this species. That hCG and LHRHa elicited different responses was not unexpected because past research in anurans has shown that the relative efficacy of these hormones is highly species specific [5]. It is important to recognise, that as for the vast majority of anuran ART studies conducted to date, our study only tested each hormone at a single dose [5]. Therefore, to more thoroughly evaluate the relative efficacy of each hormone, it will be necessary to establish spermiation responses to hCG and LHRHa across a range of doses. Testing dose response relationships was beyond the scope of the current study, but this research will be incorporated into the recovery plan for the species.

Despite our finding that LHRHa was successful at inducing spermiation, hormone treated males did not release exceptionally high concentrations of sperm. On average, LHRHa treated males released approximately 4.5 × 10^3 ^sperm per millilitre, a value that is several orders of magnitude lower than hormonally induced sperm concentrations (4.0 × 10^5 ^to 4 × 10^7 ^sperm per millilitre) previously reported for anurans [22,24,39]. There may be several explanations why sperm concentrations were comparatively low in *P. corroboree*. First, the doses used may have been too low, or too high, to induce an optimal response. Even though we used concentrations approximating those found to be effective in a broad range of frog species [18,19], LHRHa may lack potency in *P. corroboree. *Another possibility is that males were not in prime physiological condition at the time of hormone treatment. In seasonally reproducing anurans such as *P. corroboree*, individuals typically coordinate their physiological state with environmental cues, which exert their effect by stimulating gonadotropin-releasing hormone neurons at the apex of the hypothalamus-pituitary-gonad axis [45]. In nature, spermatogenetic activity in *P. corroboree *commences several months prior to the onset of breeding [46], so if environmental changes that normally take place in spring (e.g. increasing photoperiod) were not suitably replicated in the captive environment, male investment in spermatogenic activity may have been limited prior to treatment. Another explanation for low sperm yield is that *P. corroboree *is not a species that invests heavily in spermatogenesis. Theoretically, anurans should only experience strong selection for high sperm production if male's either experience a high risk of sperm competition [47], are required to fertilise large egg clutches [48], or have exceptionally high mating rates [49], but all these conditions are absent in *P. corroboree *[50,51]. In fact, testes size relative to body size in *P. corroboree *is amongst the smallest reported in the family Myobatrachidae [46,51], so assuming that testis size reflects sperm production capacity in anurans [27], male *P. corroboree *might actually be incapable of producing high sperm yields.

For females, administration of LHRHa stimulated the release of multiple batches of oocytes over a 24 hour period, indicating that hormone treatment was effective at inducing ovulation, and that final egg maturation was asynchronous [52]. However, the average percentage of females responding was low (<30%), and the average clutch size (mean = approx 15 eggs) was at the lower end of the range (16-40 eggs) previously recorded for this species [50]. The suboptimal response might mean that LHRHa lacks potency in *P. corroboree *females, as was suggested for males (see above). Indeed, the ability of LHRHa to induce ovulation is known to vary considerably between anuran species. For example, doses required to reliably stimulate ovulation in the leptodactylid frog *Eleutherodactylus coqui *are 25 times higher than needed in the Wyoming toad *Bufo baxteri *[8,17]. Quantifying dose response relationships for female *P. corroboree*, as well as for males (see above), would provide valuable insight into the potency of LHRHa in this species. An alternative explanation is that phenotypic differences between test females influenced their responsiveness to hormone treatment. For example, in a recent study on boreal toads (*Bufo boreas boreas*), Roth et al. [23] showed that female age, body size and condition were all important factors influencing the efficacy of LHRHa treatment. However, in our study, females were of similar age (4-5 yrs) and there was no relationship between female body size and number of eggs released, so it is unlikely that these factors underpinned the variable responses reported. A more plausible explanation is that unresponsive females failed to ovulate because their oocytes were immature at the time of hormone treatment [42]. This explanation seems likely because post experimental tactile inspection of unresponsive females revealed that most individuals were still carrying small to medium sized oocytes.

In nature, female *P. corroboree *undergo rapid oocyte growth 4-8 weeks before the onset of breeding [46], so assuming that similar changes take place in captivity, our treatments may not have perfectly coincided with the time when females were undergoing the final stages of oogenesis. If females were treated too early, oocytes might not have been competent for ovulation [53], but if they were treated too late, oocytes may have already commenced reabsorption [46]. Increasing egg yield in *P. corroboree *may therefore require developing techniques for reliably assessing changes in oocyte growth and development [54]. Furthermore, it may be necessary to artificially accelerate and synchronise ova maturation prior to any attempt to induce ovulation. Past work with anurans has shown that this might be achieved by repeatedly administering females with low-dose injections of gonadotropins. For example, Browne *et al. *[8] reported that two priming injections of hCG, in combination with LHRHa, significantly increased the percentage of spawning females, the number of oocytes released, and the survival of fertilised eggs in the Wyoming toad *Bufo baxteri*.

Oocyte maturation in frogs might also be enhanced via *in vitro *or *in vivo *treatment with steroid stimulants [53,55]. The process of oocyte development in anurans has been extensively studied in *Xenopus laevis *and *Rana pipiens *and it is well established that the secretion of progesterone from late-stage follicles plays a fundamental role in germinal vesicle breakdown (GVBD) and oocyte maturation [55-63]. Furthermore, there is also experimental evidence to suggest that *in vivo *administration of progesterone can accelerate oocyte maturation and increase the effectiveness of hormone treatment. Specifically, Browne et al. [42] recently reported that administration of progesterone, in combination with LHRHa, significantly improved the number and quality of hormone-induced oocytes released by the toad *Bufo fowleri*. Based on these results, incorporating steroid treatment into future ART work with *P. corroboree *might be a valuable next step towards optimising ovulatory responses in this species.

Importantly, the hormone treatment protocols we employed permitted gametes to be collected and IVF to be attempted, indicating that there is real potential for ART to assist with the captive breeding of *P. corroboree*. However, the IVF trials resulted in variable levels of fertilisation success. Variable fertilisation success may have occurred because sperm concentrations were suboptimal. In previous anuran ART studies, concentrations of sperm resulting in high fertilisation success have been within the range of 5 × 10^5 ^to 1 × 10^6 ^sperm per mL [5], but we were restricted to using concentrations of less than 2.8 × 10^2 ^sperm per mL, which may have greatly reduced the probability of gamete fusion. Alternatively, variable fertilisation success may have resulted because the osmolality of the fertilisation medium was inappropriate, as has been reported in the Australian myobatractid frog *Limnodynastes tasmaniensis *[27]. During the study all males were kept well hydrated, so if their spermic urine was too dilute, this may have significantly reduced the osmolality of the fertilisation medium. If so, sperm may have been activated soon after urine collection and lost viability before IVF was attempted. Furthermore, if osmolality was too low this could have resulted in the large egg capsules swelling too rapidly, making it impossible for individual sperm to penetrate an egg cortex. Clearly, further work will be needed to identify the primary cause of low fertilisation success. It may also be necessary to investigate alternative IVF techniques, such as intra-cytoplasmic sperm injection (ICSI). This more sophisticated IVF approach has been trialled in *Bufo arenarum *[64] and *Xenopus laevis *[65], and provides a promising solution for achieving high fertilisation success when numbers of spermatozoa are limiting [7].

Of greater immediate concern than suboptimal fertilisation, is the result that all embryos failed during early development. Early embryo failure may have occurred because hormonal induction impaired gamete viability [5]. In most studies testing the feasibility of IVF in anurans, sperm has been obtained from testes macerates, and embryo survival has been high [5,13,27-29]. However, in a recent study in which hormonally induced sperm was used to conduct IVF in the endangered toad *Bufo baxteri*, levels of embryo survival were also low [8]. These results suggest that there might be problems associated with using spermic urine to fertilize hormonally induced oocytes. Potential reasons for this are not obvious, so this is an area that may require research attention. An alternative explanation for embryo failure is that the incubation conditions employed were inappropriate for this species. Terrestrial breeding *Pseudophryne *species have extremely large and gelatinous eggs whose capsule size and surface area is primarily determined by hydration state [66]. Consequently, the amount of water present during incubation will significantly affect rates of gas exchange, and subsequently, embryo growth and survival [67]. If eggs were kept too hydrated, or were hydrated too early in development, this may have resulted in oxygen limitation within egg capsules, which in turn could have resulted in embryo mortality [67]. However, this seems unlikely because our incubation conditions were similar to those previously used to successfully rear *Pseudophryne guentheri *embryos (Silla unpublished data). As such, we suspect that there may be a more intrinsic explanation for embryo failure.

In a recent study that used a cross classified breeding design to examine genetic compatibility in *Pseudophryne bibronii*, a sister species to *P. corroboree*, we discovered that crosses made between individuals from the same population had high embryo survival, but those made between populations experienced complete early embryo failure (Byrne and Silla, unpublished data). These results indicate that terrestrial toadlets may be susceptible to high levels of genetic incompatibility, as has been reported in other Australian anurans [68,69]. Critically, the frogs used in our study were all sourced from mixed populations, so there is a real possibility that embryo failure was indeed linked to developmental problems arising from genetic incompatibility. To address this potential problem, it will be necessary to conduct future IVF trials in *P. corroboree *using individuals derived from the same source populations.

## Conclusion

In conclusion, this study demonstrated that exogenous hormones induced spermiation and ovulation in the southern corroboree frog *P. corroboree*. The hormone-treatment protocols used did not lead to exceptionally large numbers of gametes being released, but did permit the conduction of IVF, which resulted in moderate fertilisation success. Critically, however, all embryos failed during early stages of development. Embryo developmental failure may have occurred either because hormone treatment compromised gamete viability or because the incubation conditions employed were inappropriate. Alternatively, failure may have been linked to genetic incompatibility resulting from crosses being made between frogs sourced from several different populations. Additional work will be required to increase gamete yield for IVF and identify the causation of embryo failure. The findings are an important first step towards developing artificial reproductive technologies for assisting with the captive breeding of Australia's most critically endangered anuran.

## Competing interests

The authors declare that they have no competing interests.

## Authors' contributions

PGB and AJS were equally responsible for designing the experiments, coordinating and conducting the experiments, analysing the data and writing the manuscript. Both authors read and approved the final manuscript.
